# *Setd1a* Loss-of-function Disrupts Epigenetic Regulation of Ribosomal Genes via Altered DNA Methylation

**DOI:** 10.1093/schbul/sbaf091

**Published:** 2025-06-12

**Authors:** Nicholas E Clifton, Stefania Policicchio, Emma M Walker, Isabel Castanho, Matthew L Bosworth, Kirtikesav S Saravanaraj, Joe Burrage, Jeremy Hall, Emma L Dempster, Eilis Hannon, Anthony R Isles, Jonathan Mill

**Affiliations:** Department of Clinical and Biomedical Sciences, Faculty of Health and Life Sciences, University of Exeter, Exeter, United Kingdom; Department of Clinical and Biomedical Sciences, Faculty of Health and Life Sciences, University of Exeter, Exeter, United Kingdom; Department of Clinical and Biomedical Sciences, Faculty of Health and Life Sciences, University of Exeter, Exeter, United Kingdom; Department of Clinical and Biomedical Sciences, Faculty of Health and Life Sciences, University of Exeter, Exeter, United Kingdom; Division of Psychological Medicine and Clinical Neurosciences, Cardiff University, Cardiff, United Kingdom; Department of Clinical and Biomedical Sciences, Faculty of Health and Life Sciences, University of Exeter, Exeter, United Kingdom; Department of Clinical and Biomedical Sciences, Faculty of Health and Life Sciences, University of Exeter, Exeter, United Kingdom; Division of Psychological Medicine and Clinical Neurosciences, Cardiff University, Cardiff, United Kingdom; Neuroscience and Mental Health Innovation Institute, Cardiff University, Cardiff, United Kingdom; Department of Clinical and Biomedical Sciences, Faculty of Health and Life Sciences, University of Exeter, Exeter, United Kingdom; Department of Clinical and Biomedical Sciences, Faculty of Health and Life Sciences, University of Exeter, Exeter, United Kingdom; Division of Psychological Medicine and Clinical Neurosciences, Cardiff University, Cardiff, United Kingdom; Department of Clinical and Biomedical Sciences, Faculty of Health and Life Sciences, University of Exeter, Exeter, United Kingdom

**Keywords:** methylation, SETD1A, genes, DNA, ribosomes, mitochondria, schizophrenia, rare variant

## Abstract

**Background and Hypothesis:**

SETD1A, a histone methyltransferase, is implicated in schizophrenia through rare loss-of-function mutations. While SETD1A regulates gene expression via histone H3K4 methylation, its influence on broader epigenetic dysregulation remains incompletely understood. We explored the hypothesis that *SETD1A* haploinsufficiency contributes to neurodevelopmental disruptions associated with schizophrenia risk via alterations in DNA methylation.

**Study Design:**

We profiled DNA methylation in the frontal cortex of *Setd1a*^+/−^ mice across prenatal and postnatal development using Illumina Mouse Methylation arrays. Differentially methylated positions and regions were identified, and their functional relevance was examined through gene and biological annotation. We integrated these findings with transcriptomic and proteomics datasets, and assessed mitochondrial complex I activity to explore potential downstream functional effects.

**Study Results:**

*Setd1a* haploinsufficiency resulted in widespread hypomethylation of genes related to ribosomal function and RNA processing that persisted across all developmental stages. Setd1a-targeted promoter regions and noncoding small nucleolar RNAs were also enriched for differentially methylated sites. Despite the downregulation of mitochondrial gene expression, the same genes were not differentially methylated, and complex I activity in *Setd1a*^+/−^ mice did not differ significantly from controls. Genes overlapping hypomethylated regions were enriched for common genetic associations with schizophrenia.

**Conclusions:**

Our findings suggest that *SETD1A* haploinsufficiency disrupts the epigenetic regulation of ribosomal pathways. These results provide insight into an alternative mechanism through which genetic variation in *SETD1A* influences developmental and synaptic plasticity, contributing to schizophrenia pathophysiology.

## Introduction

Schizophrenia is a severe psychiatric disorder with a complex genetic basis, involving both common variants with small individual effects^1^ alongside a smaller number of rare, highly penetrant mutations that act to significantly increase risk.^2,3^ Genome-wide association studies (GWAS) have shown that many common schizophrenia risk variants localize to noncoding regulatory elements suggesting that they contribute to disease by influencing gene expression.^4^ However, some rare mutations may also exert effects through epigenetic dysregulation at the level of chromatin remodeling and broader epigenetic dysregulation, rather than direct disruption of noncoding regulatory sequences.

Among the rare, high-penetrance variants linked to schizophrenia, loss-of-function (LoF) mutations in SET Domain containing 1A (*SETD1A*), a histone methyltransferase, have been strongly implicated, conferring a 20-fold increased risk for schizophrenia.^2,5^  *SETD1A* primarily mediates histone H3K4 methylation, a modification that marks active transcriptional states and plays a key role in gene regulation during brain development, as well as cell cycle control^6,7^ and DNA repair.^8,9^ Disruptions in *SETD1A* function affect neuronal development and function.^10–14^ Given the interplay between histone modifications and DNA methylation in epigenetic regulation,^15^  *SETD1A* LoF may indirectly disrupt DNA methylation patterns, contributing to neurodevelopmental dysfunction.

We previously explored the impact of *Setd1a* haploinsufficiency on the cortical transcriptome and synaptic proteome across multiple developmental stages in mice, revealing substantial downregulation of genes involved in mitochondrial pathways,^16^ since replicated,^17^ and disruption to synaptic proteins. These *Setd1a*^+/−^ mice exhibit sensorimotor gating deficits and increased anxiety,^17^ in line with other mouse models of *Setd1a* LoF.^12,14^ However, the extent to which *Setd1a* LoF affects epigenetic regulation, particularly DNA methylation, during development remains unexplored.

DNA methylation is the best-characterized epigenetic modification, acting dynamically across development to influence gene expression via disruption of transcription factor binding and recruitment of methyl-binding proteins that initiate chromatin compaction and gene silencing.^15^ Although traditionally associated with transcriptional repression, DNA methylation influences gene expression in either direction^18^ and other genomic functions including alternative splicing and promoter usage.^19^ Recent studies have shown evidence for altered DNA methylation in the cortex from individuals with schizophrenia,^20,21^ with disease-associated variation enriched in developmentally dynamic regions of the genome. It has also been demonstrated that DNA methylation is under strong genetic control, with an enrichment of fetal brain DNA methylation quantitative trait loci colocalized to genomic regions associated with schizophrenia^22^ and evidence for genome-wide “episignatures” linked to pathogenic mutations in neurodevelopmental disorders.^23^

In this study, we profile genome-wide patterns of DNA methylation in the frontal cortex of a Setd1a^+/−^ mouse model at 5 developmental stages—embryonic day (E)14.5, E18.5, postnatal day (P)7, P35, and P70—allowing us to assess the onset and persistence of epigenetic effects caused by *Setd1a* haploinsufficiency. The selected timepoints capture key developmental processes of relevance to schizophrenia pathology, including early neurogenesis and neuronal migration, synaptogenesis, and the refinement and maturation of neuronal circuits. We identify widespread DNA methylation changes associated with haploinsufficiency persisting throughout these timepoints. By integrating these findings with transcriptomic and proteomics data, we examine whether epigenetic disruption by *SETD1A* LoF converges on pathways implicated in schizophrenia. Additionally, we assess mitochondrial complex I activity to evaluate whether previously observed transcriptomic changes correspond to functional deficits. Collectively, this work provides new insight into the developmental epigenetic consequences of *SETD1A* LoF and their relevance to schizophrenia-associated pathways.

## Methods

### Subjects and Tissue Preparation

Heterozygous *Setd1a*^*tm1d*^ (*Setd1a*^+/−^) mice on a mixed C57BL/6NTac and C57BL/6J background were generated and maintained as described previously.^16,17^ The knockout allele was generated through a knockout-first design targeting exon 4, giving premature termination of *Setd1a* transcripts and effective elimination of functional Setd1a protein from the mutant allele. This closely models the molecular effect of human *SETD1A* LoF mutations, many of which are protein-truncating variants.^2^ Brain tissue from *Setd1a*^+/−^ mice exhibits a ~50% drop in RNA and protein expression.^17^ Frontal cortex tissue was dissected from male *Setd1a*^+/−^ and wild type littermates at embryonic day 14.5 (E14.5), E18.5, postnatal day 7 (P7), P35 and P70. Tissue was snap-frozen on dry ice and stored at −80 °C. All procedures were conducted in accordance with the United Kingdom Animals (Scientific Procedures) Act 1986 (PPL 30/3375) and complied with the ARRIVE guidelines.

### DNA Methylation Profiling

Frontal cortices (*n* = 5 per genotype per timepoint) were homogenized by Dounce grinding and DNA extracted using the AllPrep DNA/RNA micro kit (Qiagen). Approximately 500 ng of DNA from each sample was treated with sodium bisulfite using the EZ-96 DNA methylation kit (Zymo Research). DNA methylation was quantified at ~285 000 sites across the genome using the Illumina Infinium Mouse Methylation array^24^ using the manufacturers’ standard protocol. Samples were pseudorandomly distributed across Illumina chips and balanced across litters to minimize batch and litter-related confounding. Matched RNA and proteomic data from the same samples were available as previously described.^16^ DNA methylation data quality control was performed using a bespoke preprocessing pipeline (https://github.com/ew367/mouseArray). In brief, methylated and unmethylated signal intensities at each position were extracted from raw IDAT files using the *ENmix* R package^25^ and converted to beta values reflecting the proportion of DNA methylation at each position. All samples achieved a minimum median methylated or unmethylated signal intensity value of 2000 with fewer than 1% of probes failing the detection *P* value threshold of 0.05, determined with *ENmix*. All samples passed a bisulfite conversion efficiency threshold of 90% (mean = 96%). Principal component analyses were used to evaluate sample clustering. Following sample QC, we performed probe filtering. 24 860 probes flagged in the Illumina manifest as exhibiting high technical variability were removed. Additional probes failing the detection threshold of 0.05 in more than 1 sample were also excluded. The resulting data matrix was quantile normalized using the *wateRmelon* R package.^26^ The final dataset included 50 samples and DNA methylation estimates for 262 086 genomic positions (247 810 autosomal sites and 14 276 sex chromosome sites).

### Cell Type Deconvolution

Reference DNA methylation data were generated from a parallel project in which we purified neuron-enriched (NeuN+ve) and glia-enriched (NeuN−ve) nuclei populations from frontal cortex tissue dissected from 30 adult (1.5–12 months) wildtype C57BL/6J mice using fluorescent activated nuclei sorting using a method optimized by our group.^27^ DNA was extracted from ~50 000 nuclei using a phenol:chloroform extraction protocol^28^ and DNA methylation was profiled and preprocessed as described above. NeuN+ve and NeuN−ve data were normalized separately. Data from 5 nuclei fractions originating from 3 animals were excluded due to failing quality control with a high proportion of sites with nonsignificant detection *P* values (Figure S1). The DNA methylation data for these samples was used to train a reference-based constraint projection deconvolution algorithm, as originally proposed by Houseman et al.^29^ to estimate the quantitative variables from bulk tissue DNA methylation profiles that capture the cellular composition of these samples. This was implemented using functions from the CETYGO package (https://github.com/ejh243/CETYGO) and methods adapted from a recent analysis of human cortex by our group.^30^ In brief the method includes the following steps: After filtering to autosomal sites, an ANOVA was used to find differences in methylation between reference cell types. Sites were ranked by their ANOVA *P* values, selecting those with significant differences (*P* < 1 × 10^−8^), focusing on the top 50 for both hypo- and hyper-methylation in each reference cell type (100 total). For each site, the average DNA methylation level for each reference cell type across all samples is used as input for the deconvolution algorithm. In addition to estimating the proportion of NeuN+ve and NeuN−ve nuclei of each bulk tissue sample, we calculated an error metric, the CETYGO score which quantifies the deviation between a sample’s DNA methylation profile and its expected profile given the estimated cellular proportions.^31^ We used this metric to evaluate the quality of the cellular composition variables across sample groups.

### Pseudo-age Scoring

Pseudo-age scoring was performed using 105 DNA methylation sites included on the Infinium Mouse Methylation array that were previously associated with age using blood samples from C57BL/6 mice 11–117 weeks old.^32^ Previous studies have shown that age estimates from epigenetic clocks are highly correlated across tissues.^33^ To derive a pseudo-age score for each WT and *Setd1a*^+/−^ sample, we used the following method:


$$\begin{array}{l} Pseudo\text{-}age\;score\;=\;\sum_{i=1}^{105}\left(s_{i}\cdot \beta_{i}\right) \end{array}$$

Where *s*_*i*_ is the slope estimated from a linear regression model between DNA methylation at site *i* and chronological age, and *β*_*i*_ is the normalized methylation beta value for probe *i*.

### Differential DNA Methylation Analysis

Relationships between genotype, age, and DNA methylation were determined using linear regression. Prior to analysis, probes mapped to chromosome 0 or probes flagged by Illumina as having altered functional performance (“MFG Change Flagged,” indicating known technical issues affecting probe reliability across batches or array versions) were removed. We further selected a subset of 187 716 “variable” probes which were defined as exhibiting a >5% methylation range across the inner 80th percentile of samples when sorted by normalized beta value. These variable probes formed the background for downstream analyses. We fitted the following linear model, with age as a categorical variable:


$$\begin{array}{lll} & normalized\;beta∼genotype\; & +age+chip\;ID+predicted\;neuronal\;proportion \end{array}$$

Chip ID was included as a covariate in all linear models to statistically adjust for batch effects. Genotype by age interactions were determined using an analysis of variance contrasting the above model with that below.


$$\begin{array}{lll} & normalized\;beta\;∼\;genotype\cdot age\; & +chip\;ID+predicted\;neuronal\;proportion \end{array}$$

Resulting *P* values were adjusted for multiple testing using the Bonferroni method with differentially methylated positions (DMPs) classified as those with *P* < 1.91 × 10^−7^.

Genes were annotated to DNA methylation sites using Illumina annotations. Gene-wide *P* values were generated by combining probe-level *P* values using the Empirical Brown’s method.^34^

In secondary analyses, genes were only annotated to DNA methylation sites located in promoter regions, using the CHIPseeker Bioconductor package.^35^

### Identification of Differentially Methylated Regions

Differentially methylated regions (DMRs) were defined as more than 1 consecutive DNA methylation site spaced less than 1500 base pairs apart, with a nominally significant (*P* < .05) main effect of genotype, and each with the same direction of effect. *P* values for probes forming a potential DMR were combined using the empirical Browns’s method^34^ and resulting DMR-wide *P* values adjusted for the number of genome-wide DMRs considered, using the Bonferroni method. Differentially methylated regions were annotated to genes based on overlap with a gene body (±1500 base pairs) based on the GRCm38 genome build.

### Pathway Analysis

Functional gene annotations were compiled from the Gene Ontology (GO) database (downloaded June 5, 2024), excluding annotations with evidence codes IEA (inferred from electronic annotation), NAS (nontraceable author statement), or RCA (inferred from reviewed computational analysis). GO terms represented by fewer than 10 genes from the statistical background (genes annotated to variable probes) were excluded from analysis. Overrepresentation of a functional term within differentially methylated genes was determined using a binomial regression model, covarying for probe density. Independence between multiple significantly enriched functional terms was determined by the iterative addition of terms to the model as covariates, prioritizing those with superior effect size. Coverage of a pathway on the methylation array was determined by calculating the proportion of genes annotated to at least 1 variable probe.

To test the significance of intersections between sets of genes not derived from annotations to methylation probes, and not necessitating the use of covariates, we used a Fisher’s exact test. For comparisons between sets of genes deriving from different omics methods, a combined background of expressed genes containing variable probes was used.

### Setd1a Target Genes

*Setd1a* target genes were identified from published chromatin immunoprecipitation and sequencing (ChIP-seq) data^13^ as previously described.^16^ Setd1a ChIP-seq peaks from 6-week-old mouse prefrontal cortex annotated to promoter regions or overlapping with H3K4me3^13^ were mapped to genes using the mm10 mouse genome assembly. Peaks mapping to zero or multiple genes were excluded. For tests of gene set enrichment, a statistical background of all genes with peaks in the ChIP-seq data^13^ was used.

### Integration With Transcriptomic and Synaptosome Protein Data

Matched transcriptomic and synaptosomal proteomic data were previously generated on the same samples.^16^ For multiomic analyses, we used 734 genes and 63 proteins differentially expressed by genotype, covarying for age effects.

### Cell Type Enrichment Analysis

Expression-weighted cell type enrichment analysis of gene sets was performed using the *EWCE* R package.^36^ Mouse cortex and hippocampus single-cell reference transcriptomes were obtained from published data.^37^ The probability of cellular enrichment was calculated using bootstrapping of random gene sets 100 000 times from a background of expressed genes containing variable methylation probes.

### Genetic Association Analysis

Genome-wide association studies summary statistics were obtained from a study of 74 776 schizophrenia cases and 101 023 controls of European, East Asian, African American, and Latino ancestry.^1^ Single nucleotide polymorphisms (SNPs) with minor allele frequency ≥1% were annotated to genes, allowing for a 35 kb upstream and 10 kb downstream window around the gene body. Single nucleotide polymorphism association *P* values were combined to derive gene-level statistics using the MAGMA (v1.10)^38^ SNP-wise mean model, adjusting for linkage disequilibrium defined by the 1000 Genomes European reference panel.^39^ To test for the enrichment of schizophrenia genetic association in each gene set, we performed a competitive gene set association analysis using MAGMA, adjusting for background enrichment. Testing of multiple gene sets was adjusted for using the Bonferroni method.

### Quantification of Mitochondrial Complex I Activity

Frontal cortices from new P70 *Setd1a*^+/−^ (3 female, 2 male) and wildtype (3 female, 3 male) mice were separated from flash-frozen brains in a sterile laminar air-flow cabinet. Left lateral frontal regions were homogenized in a 1:20 ratio with ice-cold mitochondrial homogenization buffer (50 mM Tris–HCl ph7.4, 100 mM KCl, 1.5 mM MgCl_2_, 1 mM EDTA, 50 mM HEPES, 100 mM sucrose) using a glass mortar and pestle following Dounce homogenization. The homogenate was centrifuged at 850*g* for 10 min at 4 °C (to remove cellular debris) and the resulting supernatant was centrifuged at 1000*g* for 10 min at 4 °C (to remove nuclear fraction). The following supernatant was centrifuged at 10 000*g* for 30 min at 4 °C; the resulting supernatant was discarded yielding in a mitochondrial pellet. The pellet was resuspended in the same volume of ice-cold mitochondrial homogenization buffer as used for the initial tissue homogenization. The mitochondrial extracts were stored at −80 °C.

Protein estimation was performed using the Bradford assay.^40^ BSA (BSA-Sigma Aldrich) standards of known concentrations ranging from 0.2 to 1 mg/mL, or mitochondrial extracts in homogenization buffer, were added to Bradfords reagent in a 1:4 ratio respectively in a 96-well plate (in triplicates) and incubated for 10 min at room temperature shielded from light. The absorbance was measured spectrophotometrically using a Pherastar FSX Microplate reader at 595 nm. Protein concentrations were estimated using linear regression from the known standards.

A mitochondrial complex I assay^41,42^ was performed using the Mitochondrial Complex I activity Colorimetric Assay kit (Abcam) as per the manufacturer’s instructions. A standard curve was prepared by measuring the absorbance of known concentrations of Complex I dye (0–100 nM) at 600 nm. We then undertook kinetic absorbance reading of mitochondrial extracts (0.66 μg/μL mitochondrial protein in 2 μL) and a background in a reaction mixture (Complex I buffer [1×], Decyclubiquinone [1×], Complex I dye [1×], NADH [1×]), with or without the reaction inhibitor Rotenone (1×). Readings were taken at 34 s intervals up to 374 s. Complex I enzymatic activity was calculated as follows:


$$\begin{array}{lll} & Net\;comp\;I\;activity\;(mU/\mu g)\; & =\left(\frac{{\left(\Delta C\right)}_{rotenone^{-}}}{\left(\Delta t+p\right)\cdot D}\right)-\left(\frac{{\left(\Delta C\right)}_{rotenone^{+}}}{\left(\Delta t+p\right)\cdot D}\right) \end{array}$$

Where *C* is reduced complex I dye concentration (derived from standard curve), *t* is time, *p* is the mitochondrial protein concentration and *D* is the dilution factor.

Complex I activity was compared between *Setd1a*^+/−^ and wildtype mice using a 2-way ANOVA with post hoc Šidák’s multiple comparisons test.

### RNA Isolation and qPCR

Complementary-DNA (cDNA) was generated from RNA isolated from cytosolic fractions of P7 Setd1a^+/−^ mouse cortex (5 per genotype)^16^ and used in quantitative polymerase chain reaction (qPCR) assays to ascertain the abundance of *Snord83b*, *Snord53a*, and *Snord34*. RNA isolation was performed using the Qiagen RNeasy Mini Kit-74104 with RNA resuspended in nuclease-free water. Complementary-DNA synthesis was performed using the Maxima First strand cDNA Synthesis Kit for RT-qPCR (ThermoFisher-K1641).

The qPCR reaction mixes for each probe, including 2 house-keeping genes (*Gapdh* and *Hprt1*) were made using the SSO advanced universal SYBR-green Supermix (Biorad-1725270), their respective forward and reverse primers (Table S10) and nuclease-free water. The qPCR reactions were run in duplicates for each sample per probe on a StepOne realtime qPCR system (ThermoFisher). Each reaction well comprised 10 μL of the qPCR reaction mix and 5 μL of sample cDNA to a normalized concentration. Nuclease-free water was used as a negative control.

The qPCR reaction curve consisted of a holding stage (95 °C for 30 s), cycling stage (40 cycles; 95 °C for 15 s then 60 °C for 60 s), and a melt curve stage (95 °C for 15 s then 65 °C for 60 s then 95 °C for 15 s). The resulting data were analyzed using Welch’s unpaired *T*-test.

## Results

### Methylation-derived Predictions of Cell-type Proportion and Epigenetic Age by Genotype

**Figure 1.**
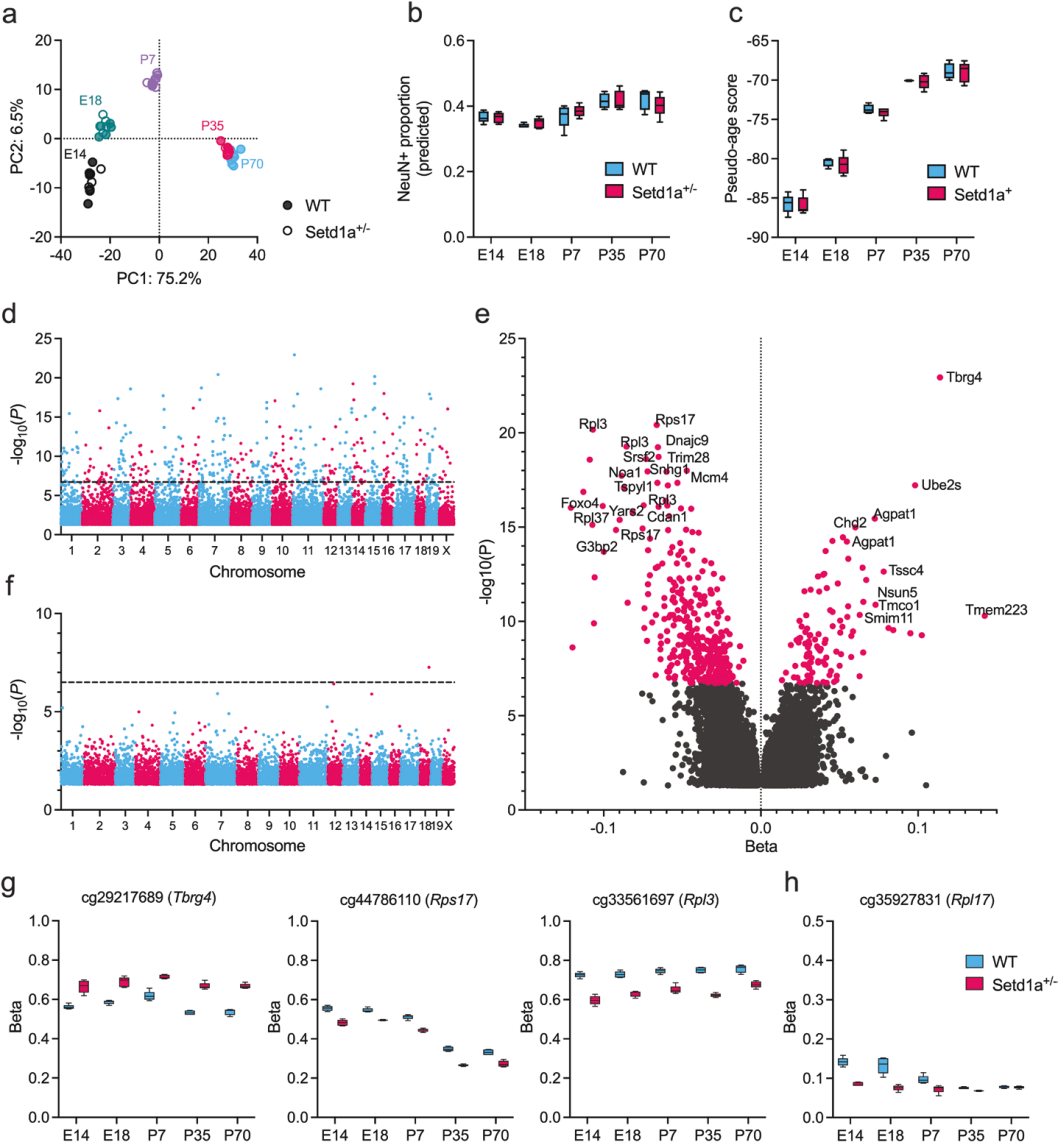
DNA Methylation in Frontal Cortex of *Setd1a*^+/−^ Mice at 5 Developmental Stages. (A) Principal component analysis (PCA) of DNA methylation data from all *Setd1a*^+/−^ and wildtype mice profiled across developmental stages (E14.5, E18.5, P7, P35, P70; *n* = 5 per genotype per age), demonstrating clustering by age. Shown are the first and second principal components derived from normalized beta values. (B) Predicted neuronal cell type proportions by genotype across all samples. Predictive models were based on DNA methylation in NeuN+ve and NeuN−ve nuclei isolated from independent samples of wildtype mouse frontal cortex. (C) DNA methylation-derived pseudo-age scores across sample groups, showing a strong correlation with chronological age. No effect of genotype was observed. Age-associated DNA methylation sites identified previously.^32^ (D) Manhattan plot displaying the significance of differential DNA methylation across chromosomes on the Illumina Infinium Mouse Methylation array from genotype comparisons, adjusting for age. The dotted line indicates a threshold for genome-wide significance after Bonferroni correction. Colors delimit chromosomes. (E) Volcano plot showing gene annotations to top differentially methylated positions (DMPs). Displayed is the relationship between effect size (Beta) and −log_10_(*P*) from genotype contrasts, adjusting for age. (F) Manhattan plot displaying the significance of differential methylation in genotype-by-age interaction analysis. Only 1 site (cg35927831 annotated to Rpl17) surpassed the Bonferroni threshold for significance. (G) Differential DNA methylation at the 3 top-ranked genotype DMPs annotated to *Tbrg4*, *Rps17*, and *Rpl3*. Shown are the probe-level methylation beta values by age and genotype. (H) Genotype-by-age interaction effect on DMP cg35927831 annotated to *Rpl17*. E14, embryonic day 14; E18, embryonic day 18; P7, postnatal day 7; P35, postnatal day 35; P70, postnatal day 70; WT, wildtype.

Following preprocessing and stringent quality control (Figure S2), our final dataset included DNA methylation data for 262 112 sites quantified in prefrontal cortex tissue from *Setd1a*^+/−^ and wildtype mice at 5 developmental stages (*N* = 5 per genotype per stage). Following dimensionality reduction, the first 2 principal components showed clear clustering of samples by age (Figure 1A).

We used cell type deconvolution to predict the effect of genotype and age on the proportion of neuronal cells in our samples. Fluorescent activated nuclei sorting-derived neuronal (NeuN+ve) and nonneuronal (NeuN−ve) DNA methylation data from independent cortex tissue samples were used to calculate weights for cell type deconvolution (Figure S1). Deconvolution accuracy, indicated by the CETYGO score (Figure S2C), was weaker for embryonic and P7 samples, likely reflecting the adult origin of the reference dataset. Predicted cell type proportions showed variation across age groups (*β* = 8.6 × 10^−4^ per day, *P* = 8.36 × 10^−5^; Figure 1B), but no significant difference between genotype groups in the proportion of neuronal cells was identified (*β* = −5.4 × 10^−4^, *P* = .73).

As a proxy for the effects of genotype on the molecular mechanisms underlying biological aging, we evaluated epigenetic age. 105 age-associated probes identified in a previous study^32^ were used to calculate a pseudo-age score for each sample. DNA methylation-derived pseudo-age scores were strongly associated with mouse chronological age (*β* = .19 per day, *P* = 1.3 × 10^−6^; Figure 1C), but no effect of genotype on pseudo-age score was observed (*β* = −.071, *P* = .96).

### Differentially Methylated Positions Associated With *Setd1a* Genotype

We compared levels of DNA methylation across the genome in frontal cortex between *Setd1a*^+/−^ mice and wild type controls, adjusting for age, batch (ie, array chip), and predicted neuronal proportion. After Bonferroni correction for multiple testing, we identified 356 hypomethylated DMPs (*P* < 1.9 × 10^−7^) and 105 hypermethylated DMPs in *Setd1a*^+/−^ tissue compared to wildtype mice (Figure 1D; Table S1). For functional interrogation, DMPs were annotated to genes as per Illumina annotations (Figure 1E). The 3 probes exhibiting the most significant between-group differences in DNA methylation (cg29217689, *P* = 1.2 × 10^−23^; cg44786110, *P* = 3.8 × 10^−21^; cg33561697, *P* = 6.8 × 10^−21^) were annotated to transforming growth factor beta regulator 4 (*Tbrg4*), ribosomal protein S17 (*Rps17*), and ribosomal protein L3 (*Rpl3*), respectively (Figure 1F). Through aggregation of raw probe-level *P* values to corresponding genes and adjusting for the number of genes annotated to at least 1 probe, we identified 307 differentially methylated genes (Table S2) with the top-ranked genes being RNA binding motif protein 15B (*Rbm15b*; *P* = 1.8 × 10^−45^), *Rpl3* (*P* = 2.3 × 10^−32^), and flavin adenine dinucleotide synthetase 1 (*Flad1*; *P* = 1.7 × 10^−31^).

In a genotype-by-age interaction analysis, we identified 1 DMP (cg35927831 annotated to Rpl17; *F* = 17.9, *P* = 5.5 × 10^−8^) that exhibited a significant interaction effect (Figure 1F, H; Table S1). The lack of further interactions suggests that the effects of *Setd1a* LoF on DNA methylation did not systematically differ by age, mirroring our previous gene expression results^16^ and indicating that the changes identified become manifest early in development and remain stable across the life-course.

**Figure 2.**
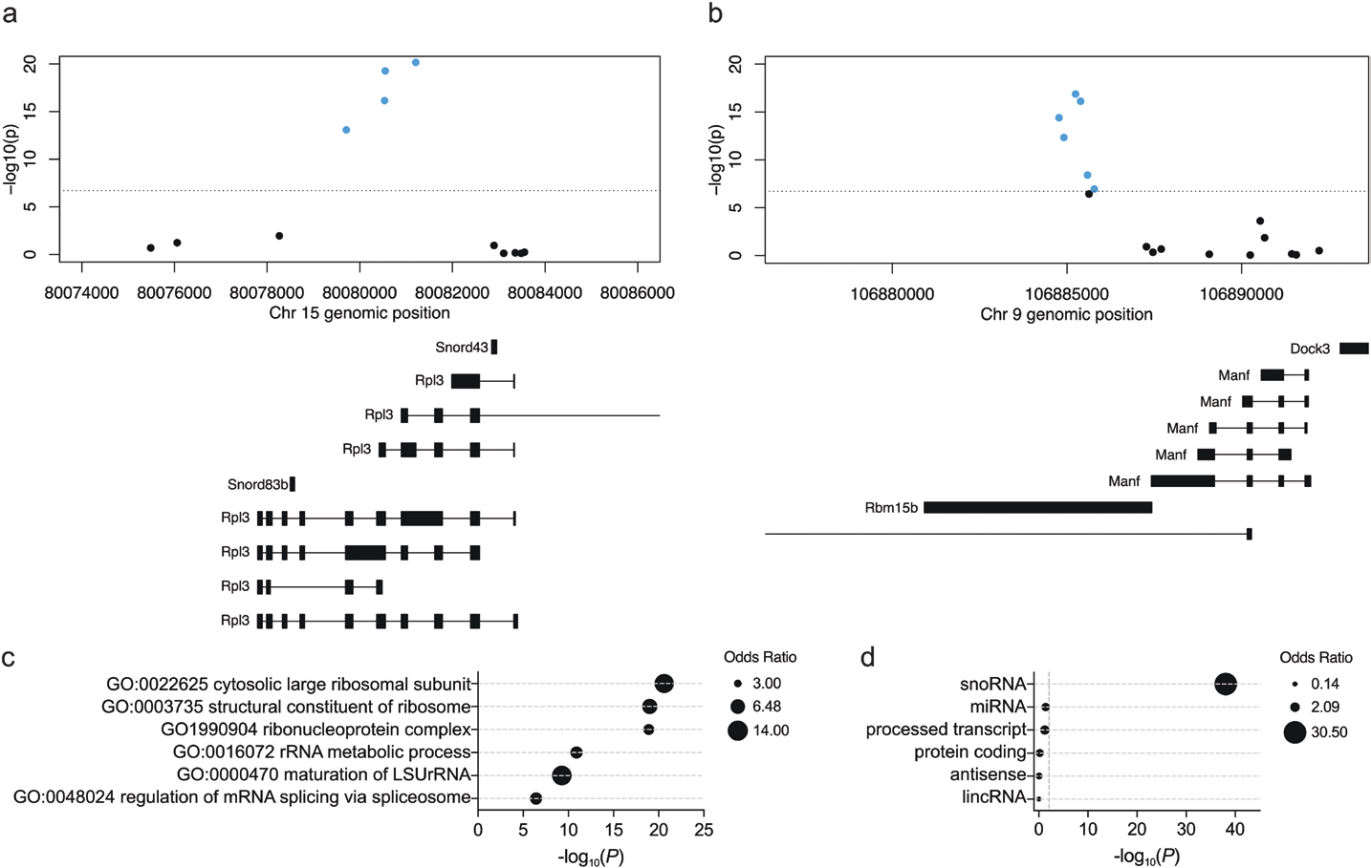
Gene and Functional Annotations of Differentially Methylated Positions Associated With Setd1a Genotype. (A, B) Two example differentially methylated regions (DMRs) identified in *Setd1a*^+/−^ mice annotated to genes including *Rpl3*, *Snord83b*, *Manf*, and *Rbm15b*. Blue dots show significantly hypomethylated sites compared to wildtype, adjusting for age. Below each plot is displayed exon positions for known gene transcripts. (C) Pathway enrichment analysis highlights significant enrichment of ribosomal structure and RNA processing functions among differentially methylated genes. Shown is the −log_10_(*P* value) in a binomial regression analysis, covarying for probe density. The size of the dots is determined by the unadjusted odds ratio. (D) Enrichment analysis using gene biotypes showed an overrepresentation of small nucleolar RNAs (snoRNAs) in differentially methylated genes.

We identified 410 DMRs between genotype groups annotated to 646 unique genes (including 484 protein-coding genes; Table S3). The top-ranked DMR was annotated to *Rpl3* and small nucleolar RNA, C/D Box 83B (*Snord83b*; chr15:80078264-80081207; *P* = 2.5 × 10^−34^) and the next most significant DMR annotated to mesencephalic astrocyte derived factor (*Manf*) and *Rbm15b* (chr9:106884775-106885781; *P* = 1.9 × 10^−33^; Figure 2A, B). These were subdivided into 445 genes (319 protein-coding) containing hypomethylated regions and 206 genes (170 protein-coding) containing hypermethylated regions (5 genes contained both hypomethylated and hypermethylated regions). Genes annotated to DMRs overlapped with 73% of genes deemed significantly differentially methylated via probe-level aggregation.

**Figure 3.**
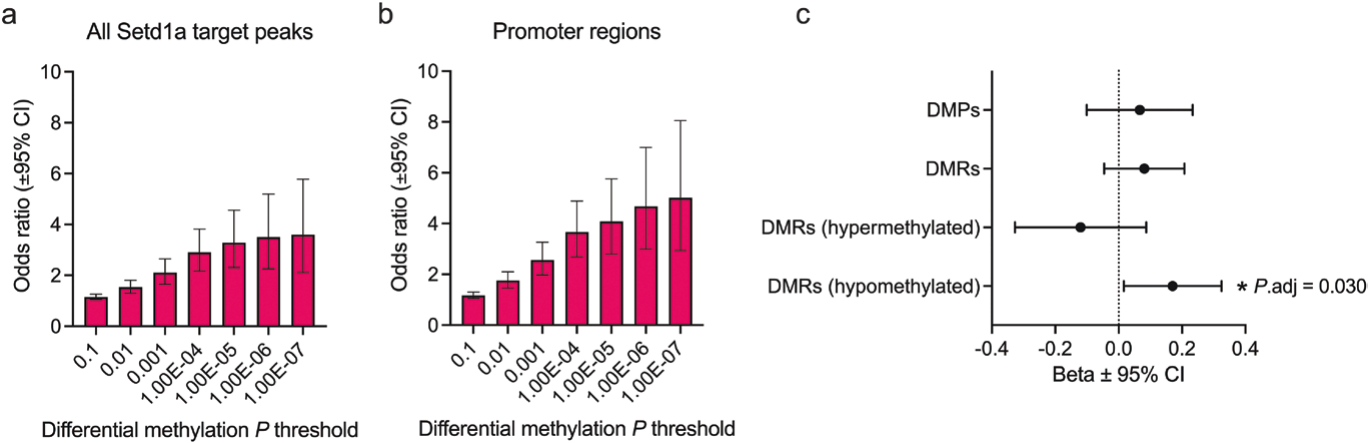
The overlap of differentially methylated sites with Setd1a target regions and schizophrenia-associated regions from GWAS. (A) The enrichment of Setd1a target regions for differentially methylated positions (DMPs) increases with the confidence of differential methylation. Shown is the odds ratio ± 95% confidence interval (CI) from Fisher’s exact test of the overlap between Setd1a target peaks from ChIP-seq^13^ and DMPs defined by 6 *P* value thresholds. All genes represented by the ChIP-seq data were used as a statistical background. (B) The enrichment of promoter-specific Setd1a target regions for DMPs across 6 *P* value thresholds. (C) The enrichment of schizophrenia GWAS associations in human homologues of genes annotated to DMPs or differentially methylated regions (DMRs).

Pathway analyses were based on differentially methylated genes defined by gene-level analyses, then divided by direction of effect using annotations from regional analyses. Differentially methylated genes were enriched for functions principally related to ribosome structure and function, and RNA processing (Figure 2C; Table S4). These functional annotations were driven by genes overlapping hypomethylated regions (Figure 3A; Table S5), while the smaller number of genes containing hypermethylated regions were modestly enriched for 3 functional terms related to glycogen metabolism (Figure 3B; Table S6). Since DNA methylation in regulatory regions is known to influence gene expression, we repeated the annotation of differentially methylated sites, restricting it to promoter regions. Differentially methylated genes identified in this way were broadly enriched for the same functional categories as the primary set (Table S7), suggesting that the regulatory activity of these biological pathways, particularly ribosomal, is altered by *Setd1a* LoF. Mitochondrial pathways previously shown to be enriched for differentially expressed genes in these tissues^16^ were not associated with differential methylation at the sites provided on the array, after correcting for multiple comparisons (eg, GO:0005739 *Mitochondrion*: 84.9% array coverage, odds ratio = 1.14, *P*.bonf = 1.0).

Due to the restriction of the gene ontology database to predominantly protein-coding genes, our pathway analysis excluded differentially methylated noncoding genes. By performing gene set enrichment by biotype, we identified a major overrepresentation of small nucleolar RNAs (snoRNAs) among the differentially methylated genes (odds ratio = 30.5, *P*.bonf = 1.3 × 10^−37^; Figure 2D; Table S8). Since many snoRNA host genes encode ribosomal proteins,^43^ and the Infinium mouse methylation array has poor coverage of snoRNAs in general, we hypothesized that this result was driven by the differential methylation of biological pathways consisting of overlapping protein-coding genes (Table S9). After adjusting for snoRNAs sharing DMPs with significantly overlapping pathways, we found that the enrichment of differentially methylated genes for snoRNAs was attenuated but remained significant (*P* = 3.6 × 10^−12^). Likewise, the top ribosomal pathway, *GO:0022625 cytosolic large ribosomal subunit*, remained strongly enriched in differentially methylated genes after adjusting for overlapping snoRNAs (*P* = 4.6 × 10^−12^). Overall, DNA methylation changes induced by *Setd1a* LoF were preferentially localized within genes encoding proteins with roles in RNA processing and translation, and noncoding snoRNAs.

To further explore the relationship between histone methylation by Setd1a and alterations in DNA methylation, we investigated the intersection between DMPs and previously proposed Setd1a-target regions, derived from ChIP-seq.^13^ Of 461 DMPs, 21 overlapped a Setd1a target promoter region, significantly more than expected based on a background of all variable methylation probes (Fisher’s exact test: odds ratio 5.26, *P* = 2.8 × 10^−9^). The strength of this overlap increased with the significance threshold used to define DMPs (Figure 3A, B). We found that 35% of the genes annotated to DMPs overlapped with Setd1a target promotor regions (Fisher’s exact test: odds ratio = 1.39; *P* = .0098). Furthermore, in pathway analysis, genes targeted by Setd1a were enriched for functions related to protein translation and RNA processing (eg, GO:0006412 *Translation*, odds ratio = 2.00, *P*.bonf = 7.7 × 10^−10^; GO:0003723 *RNA binding*, odds ratio = 1.76, *P*.bonf = 3.0 × 10^−10^), among others. These findings highlight a clear relationship between Setd1a target sites and differential DNA methylation of the same regions, providing additional validation of differential epigenetic regulation of these genes in *Setd1a*^+/−^.

### Schizophrenia Genetic Association of Differentially Methylated Genes

We tested the hypothesis that genes with disrupted DNA methylation profiles associated with *Setd1a* LoF are themselves associated with schizophrenia genetic risk through common variation in GWAS. Following adjustment for linkage disequilibrium and background association in human homologous genes containing variable probes, we found that genes annotated to hypomethylated regions were significantly enriched for schizophrenia association (*β* = .17, *P*.bonf = 0.030). Genes annotated to hypermethylated regions (*β* = −.12, *P*.bonf = 1.0), or differentially methylated genes as a whole (*β* = 0.081, *P*.bonf = 0.21) were not significantly associated (Figure 3C).

### Integrating DNA Methylation Data With Gene Expression and Proteomic Data

**Figure 4.**
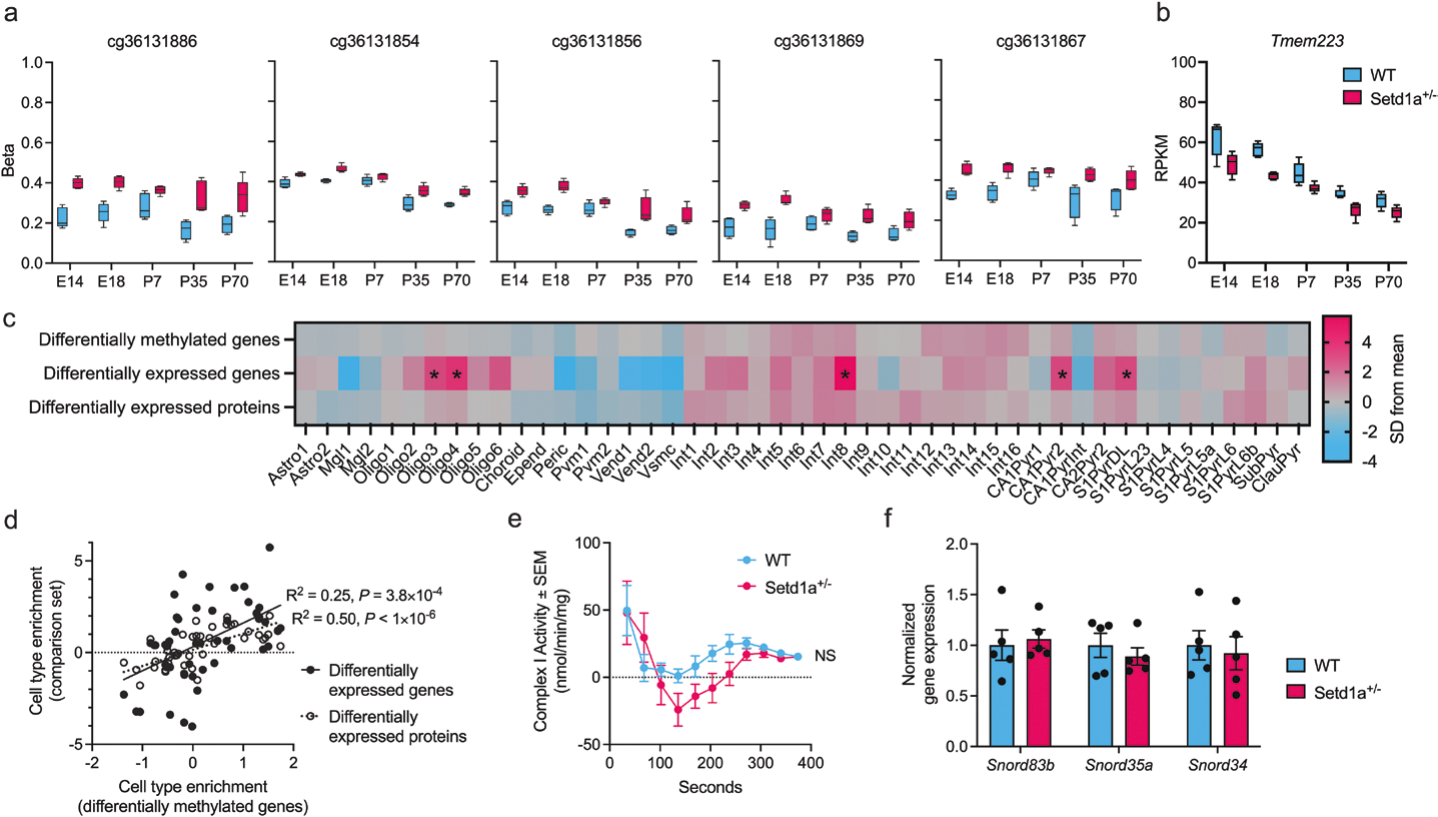
Integration of DNA Methylation With Gene Expression and Proteomic Data Generated on the Same Samples. (A) Five DMPs annotated to *Tmem223*, a highly ranked gene in multiomics analysis. Each box plot shows the distribution of beta values across genotype and age at a methylation site. Each site shows increased methylation in *Setd1a*^+/−^ frontal cortex. (B) *Tmem223* RNA is significantly downregulated in *Setd1a*^+/−^ frontal cortex across all timepoints. (C) Heatmap showing results from cell type enrichment analysis of differentially methylated genes, differentially expressed genes, and differentially expressed proteins (synaptosome), using single-cell transcriptomic data.^37^ Transcriptomics and proteomics data were published previously.^16^ Colours indicate the strength of enrichment for genes with increased (red) or decreased (blue) expression in a particular cell type, given as the number of standard deviations (SD) from the mean. Asterisks indicate significant cell type enrichment. Details of the cell type definitions can be found in the original study.^37^ (D) The relationship between cell type enrichment (SD from mean) of differentially methylated genes and either differentially expressed genes or differentially expressed proteins. (E) Mitochondrial complex I activity assay performed on P70 *Setd1a*^+/−^ (*n* = 5) and wildtype (*n* = 6) frontal cortex. Readings were taken at 34 s intervals. Shown is the Complex I enzymatic activity calculated from the net change in absorbance at 600 nm, which reflects the reduction of NADH, compared to the previous measurement. (F) Quantification of small nucleolar RNA (snoRNA) expression (*Snord83b*, *Snord53a*, *Snord34*) in *Setd1a*^+/−^ and wildtype frontal cortex (*n* = 5 per group) via qPCR. Bars show the mean ± standard error. E14, embryonic day 14; E18, embryonic day 18; P7, postnatal day 7; P35, postnatal day 35; P70, postnatal day 70; WT, wildtype; RPKM, reads per kilobase of transcript per million mapped reads; NS, nonsignificant; SEM, standard error of the mean.

We previously observed differential expression of genes and proteins between *Setd1a*^+/−^ and WT frontal cortex using the same samples, persisting across developmental stages.^16^ On the Illumina methylation array, 90.2% of genes exhibiting differential expression in RNA sequencing were represented by at least 1 probe and 84.5% contained at least 1 variable probe. Of 734 differentially expressed genes, 23 were also annotated to DMPs (odds ratio = 2.52, *P* = 1.5 × 10^−4^) and 48 overlapped with DMRs (odds ratio = 2.85, *P* = 3.4 × 10^−9^). Shared genes include the mitochondrial ribosome gene, transmembrane protein 223 (*Tmem223*) which exhibited hypermethylation across 5 sites (*P *= 9.3 × 10^−14^; Figure 4A) and decreased gene expression in *Setd1a*^*+/*−^ (Figure 4B).

Given the role of synaptic pathways in schizophrenia pathophysiology and prior evidence of their disruption in mouse models of *Setd1a* LoF,^13,14^ we previously compared the protein composition of synaptosomes taken from *Setd1a*^*+*/−^ and WT tissue.^16^ Of 63 genes with differential protein expression in synaptosomes, none had an annotated DMP while only 4 overlapped with a DMR associated with genotype (odds ratio = 2.52, *P* = .085).

To further evaluate the biological convergence between differentially methylated or expressed genes, or differentially expressed proteins, associated with *Setd1a* LoF we examined their enrichment for cell-specific expression using published mouse single-cell transcriptomics data.^37^ Differentially expressed genes were associated with interneuron subtype 8 (*Z* = 5.73, *P.bonf* < 1.0 × 10^−5^), 2 oligodendrocyte lineage subtypes (*Oligo4*: Z = 4.26, *P.bonf* = 0.0034; *Oligo3*: *Z* = 3.58, *P.bonf* = 0.022), hippocampal CA1 pyramidal neuron type 2 (*Z* = 3.60, *P.bonf* = 0.021), and deep layer cortical pyramidal neurons (*Z* = 3.54, *P.bonf* = 0.029; Figure 4C). While differentially methylated genes or differentially expressed proteins from frontal cortex were not associated with any specific cell type, the overall pattern of relative expression across cell types was correlated between the 3 omics datasets (Figure 4D).

### Mitochondrial Complex I Activity

We previously observed that mitochondrial complex I genes were underexpressed in *Setd1a*^+/−^ cortex compared to wildtype tissue.^16^ This effect was observed throughout development, including a major effect at P70. Building on this observation, we hypothesized that complex I activity might be impaired by this mutation. Mitochondrial complex I activity in P70 frontal cortex was quantified at regular intervals with and without the presence of the inhibitor rotenone, using a colorimetric assay. No overall change in Complex I activity between genotypes was observed (*F*(1,9) = 3.38, *P* = .099; Figure 4E).

### Quantification of Small Nucleolar RNA Expression

Due to fragment size selection, our previous transcriptome-wide analysis was limited in its ability to detect and quantify very short transcripts including snoRNAs.^16^ Our observation that DMPs are enriched in snoRNAs led us to hypothesize that *Setd1a* LoF may induce differential expression of these snoRNAs. We selected 3 snoRNAs annotated to highly significant DMPs and quantified their expression in the cytosolic fraction from wildtype and *Setd1a*^+/−^ frontal cortex at P7 when clear changes in the methylation of these snoRNAs were observed. We observed no effect of genotype on the expression of *Snord83b* (*t* = 0.35, *P* = .74), *Snord35a* (*t* = 0.75, *P* = .48), or *Snord34* (*t* = 0.36, *P* = .73; Figure 4f), suggesting that the effects on DNA methylation were independent of the expression of these genes. However, we cannot rule out that the expression of other snoRNAs not quantified here was affected by genotype.

## Discussion

In this study, we explored the consequences of Setd1a haploinsufficiency on DNA methylation in mouse frontal cortex across development and integrated these results with transcriptomic and proteomic datasets generated from the same samples. We report that *Setd1a* LoF leads to significant epigenetic dysregulation, particularly within genes related to ribosomal function and RNA processing, and in snoRNAs. These findings provide new insights into the role of *SETD1A* in maintaining epigenetic integrity, and in the regulation of genes that are crucial for cellular metabolism and neuronal function.

A key outcome of this study is the identification of substantial hypomethylation of genes encoding ribosomal proteins in the frontal cortex of *Setd1a*^+/−^ animals. This was also reflected in regional analysis of differential methylation. Genotype-associated differences became manifest early in development and were maintained across all developmental stages examined. Given the function of Setd1a in activating chromatin through histone methylation, its reduction could impair recruitment of DNA methylation machinery at affected loci, thereby causing hypomethylation at these sites.

Ribosomal pathways, or those more broadly related to protein synthesis, have been implicated in other recent omics studies of schizophrenia and associated genetic variants. Like *SETD1A*, *GRIN2A*, and *AKAP11* contain mutations which in humans confer substantial risk to schizophrenia and other psychiatric or neurodevelopmental disorders through rare variants.^2,3,44^ Mice carrying LoF mutations in either *Grin2a* or *Akap11* displayed altered expression of ribosomal proteins in synaptic fractions.^45,46^ Disruption to these pathways in *Grin2a* deficient mouse hippocampus was also reflected at the transcriptome level.^46^ Aryal et al. showed that ribosomes were similarly disrupted in DLPFC synapses from patients,^45^ while a second study presented evidence that DLPFC pyramidal neurons from patients with schizophrenia exhibited altered RNA expression of genes involved in protein translation.^47^ Interestingly, each of these studies observed parallel disruption to mitochondrial pathways,^45–47^ which we found altered in *Setd1a*^+/−^ cortical transcriptomes previously.^16^

Unlike the gene expression data from our previous study of this model,^16^ we observed that DNA methylation in autosomal mitochondrial genes was unaffected by genotype. This shift toward ribosomal disruption suggests that *Setd1a* may play a more prominent role in the regulation of translational machinery than previously understood. While disruptions to mitochondrial dysfunction may play an important role in conferring risks of the variant, it is possible that the primary epigenetic effect of *SETD1A* loss is on ribosomal function, which could indirectly impact mitochondrial activity at the level of protein synthesis. However, these proposed links remain speculative and lack direct experimental validation. Further studies using ribosome profiling or broader assessments of metabolic functions will be necessary for testing these hypotheses. Alternatively, there may be additional alterations in DNA methylation relevant to the regulation of mitochondrial genes that were not captured by the approach used in this study. The Illumina methylation array covers a subset of DNA methylation sites, and it is likely that some genotype-associated differences could have been missed. Notably, certain classes of transcripts such as snoRNAs and mitochondrial genes had restricted coverage, which may have led to an underestimation of their methylation differences. Similarly, reduced coverage of pathways identified as significantly differentially expressed at the transcriptomic level may contribute to the modest overlap between methylation and expression changes. Secondly, *Setd1a*^+/−^ could have effects on non-CpG methylation or other DNA modifications including DNA hydroxymethylation, both of which play important roles in neurodevelopment and synaptic plasticity,^48,49^ and which were undetected by our approach. Alternative genome-wide approaches, such as bisulfite or nanopore sequencing,^50^ would provide greater resolution to fully characterize the epigenetic impact of Setd1a haploinsufficiency.

Differential methylation of sites annotated to ribosomal genes was not mirrored by detectable changes in the expression of these same genes.^16^ Tight regulation of ribosome biogenesis^51^ may lead to resistance of cell-wide transcriptional shifts, with compensatory mechanisms masking their differential regulation. Subtle, transient, or context-specific changes in expression could be sufficient to exert a cellular phenotype while not manifesting as a statistically detectable difference from short-read RNA sequencing. Secondly, their altered epigenetic regulation could enact differential transcript usage or other post-transcriptional effects instead of quantitative changes in expression, perhaps leading to ancillary effects on mitochondrial pathways. The use of long-read sequencing may provide the insight needed here, through enabling transcript-level analysis. Notably, genes targeted by Setd1a protein via promoter regions^13^ were enriched for biological pathways related to both mitochondrial^16^ and ribosomal activity.

Our data also show that genes annotated to differentially methylated sites in *Setd1a*^+/−^ mice are enriched for snoRNAs. While many of these are hosted within ribosomal protein genes, snoRNAs were independently associated with methylation changes. Considering the role of snoRNAs in the regulation of ribosomal RNA,^43^ these results serve to further highlight the tight coupling between ribosomal gene regulation and epigenetic changes in the context of *SETD1A* LoF. It is important to note, however, that the coverage of snoRNAs on the DNA methylation array is relatively limited—with a focus on CpG-rich promoter regions—and to more clearly establish the effects of *Setd1a* disruption on the epigenetic regulation of snoRNAs, a genome-wide assessment of DNA methylation is required.

Alterations in the epigenetic regulation of ribosome function and, consequentially, changes to the expression of mitochondrial genes, could influence synaptic function and ultimately contribute to the synaptic and behavioral phenotypes observed in *Setd1a*^+/−^ animals previously. A recent study using the same *Setd1a*^+/−^ mouse model presented evidence that these animals exhibit increased anxiety-related behaviors and sensorimotor gating deficits.^17^ More broadly, mice with *Setd1a* haploinsufficiency have been reported to display deficits in axonal branching, spine formation, and synaptic vesicle exocytosis, decreased spine density, as well as hyperactivity, impaired social behavior, and deficits in working memory and sensorimotor gating.^12–14^

The integration of DNA methylation with existing transcriptomic data from the same samples^16^ revealed a modest overlap between differentially expressed and differentially methylated genes. Specifically, we identified 23 genes that were both differentially expressed and methylated, including *Tmem223*, a gene encoding a mitochondrial ribosomal protein, which exhibited increased methylation coupled with decreased expression. Altered regulation of brain mitochondrial ribosomes in schizophrenia was reported recently.^52^ The relative directionality of changes in DNA methylation and transcript expression varied across the 23 genes, highlighting the complexity of epigenetic regulation beyond canonical mechanisms of promoter-based gene repression. No genes or gene products were consistently altered in *Setd1a*^*+/*−^ across all omics methods studied, further underscoring the complexity of the molecular phenotypes and the relationships between these markers of gene activity. Differences in power across omics methods and their varying coverage may also play a role. Expression-weighted cell type enrichment analysis gave evidence that *Setd1a* haploinsufficiency may have a preferential impact on genes expressed in specific cell populations, including pyramidal and interneuron subtypes, and oligodendrocytes. However, more definitive insight will require cell-type-specific methylation profiling across brain regions and developmental stages.

The relative lack of significant genotype-by-age interactions in the DNA methylation data, coupled with the consistency of ribosomal gene dysregulation across timepoints, indicates that the epigenetic impact of *Setd1a* LoF is initiated early in development and persists throughout various developmental stages. This temporal stability suggests that *Setd1a* haploinsufficiency creates a stable, lasting epigenetic signature that could prime specific genes for later disruption. As the brain matures and neural circuits develop, these early epigenetic changes may become more critical under conditions requiring increased translation or synaptic plasticity, such as during periods of learning, memory formation, or response to neurodevelopmental stressors. These mechanisms could explain the increased risk for schizophrenia and broader neurodevelopmental disorders conferred by genetic variation in *SETD1A*.

Genes overlapping hypomethylated regions in *Setd1a*^+/−^ mice show significant enrichment for schizophrenia genetic associations, indicating that *SETD1A* haploinsufficiency potentiates epigenetic vulnerability at genomic loci already predisposed to neurodevelopmental dysregulation. While transcriptome changes caused by *Setd1a* LoF were stable across timepoints, these genes were not enriched for schizophrenia association.^16^ This suggests that epigenetic alterations in *Setd1a*^+/−^ converge with biological systems influenced by schizophrenia-linked common variation, even if not directly mirrored in development-wide gene expression. Ribosomal pathways, enriched for hypomethylation in this study, were not themselves genetically associated with schizophrenia in case–control studies.^1,2^ However, the shared involvement of ribosomal and mitochondrial pathways, especially within the synaptosome,^16,45,46^ may be indicative of disruption to local translation, which has been linked to schizophrenia and autism spectrum disorders through genetic associations.^53^ Local translation, crucial for synaptic plasticity, relies on the cooperation between ribosomes and mitochondria, demanding high energy to drive the rapid synthesis of proteins.^54,55^ Our results therefore provide insight into an alternative mechanism by which genetic variation in neuropsychiatric disorders, including schizophrenia and autism, may converge on disruptions in local translation and synaptic plasticity.

In conclusion, our study demonstrates that Setd1a haploinsufficiency is associated with significant differences in DNA methylation, particularly in regulatory regions annotated to genes involved in ribosomal biogenesis and RNA processing, providing a potential mechanistic link between *SETD1A* mutations and schizophrenia risk. Further studies are needed to determine how these epigenetic changes interact with other cellular processes and contribute to mitochondrial and synaptic phenotypes, and ultimately neurodevelopmental deficits.

## Supplementary Material

Supplementary material is available at https://academic.oup.com/schizophreniabulletin.

sbaf091_suppl_Supplementary_Figures_S1-S3_Table_S10

sbaf091_suppl_Supplementary_Tables

## Data Availability

Raw and processed data are available at the Gene Expression Omnibus (accession GSE295008).
