# Supplementary material for: Setd1a Loss-of-function Disrupts Epigenetic Regulation of Ribosomal Genes via Altered DNA Methylation
Source: Schizophr Bull. 2025 Jun 12;52(2):sbaf091. doi: 10.1093/schbul/sbaf091 (PMC12996923; doi:10.1093/schbul/sbaf091)
Supplement: sbaf091_suppl_Supplementary_Figures_S1-S3_Table_S10 [file sbaf091_suppl_supplementary_figures_s1-s3_table_s10.docx]

**Supplementary Figure S1** DNA methylation in NeuN+ve and NeuN-ve nuclei used for cell type deconvolution. (a) Median methylated (M) and unmethylated (U) intensity from NeuN+ve and NeuN-ve nuclei populations purified from adult wildtype mouse cortex. Dotted lines indicate the minimum threshold for passing quality control; samples below these lines were excluded. (b) Methylation beta value distributions in NeuN+ve and NeuN-ve populations. (c) The relationship between the first two principal components of normalised methylation beta values. Samples cluster distinctly by cell type.

**Supplementary Figure S2** DNA methylation profiles in *Setd1a*^+/-^ and wildtype (WT) cortex across five developmental stages. (a) Median methylated (M) and unmethylated (U) intensity per sample, coloured by genotype. (b) Methylation beta value distributions per sample, coloured by genotype. (c) Boxplot showing CETYGO scores by sample group, reflecting the predictive power of the cell type deconvolution model for those samples.

**Supplementary Figure S3** Functional annotations of differentially methylated regions (DMRs). (A) Pathways independently enriched for genes annotated to hypomethylated regions. Shown is the -log_10_(*P*-value) in a binomial regression analysis, covarying for probe density. Pathways passing Bonferroni correction were prioritised using iterative refinement. The size of the dots is determined by the unadjusted odds ratio. (B) All pathways enriched for genes annotated to hypermethylated regions following Bonferroni correction.

|  | **Forward Primer (5’- 3’)** | **Reverse Primer (5’- 3’)** |
| --- | --- | --- |
| **Snord 83b** | GAGGCTCTGGAATGTGCGCTG | ATTCCTCAGTCTCTCTCGGGC |
| **Snord 53a** | ACATGATGTTCTTATTCTCACGAT | CATCAGCTTAGCCTTTGGCAT |
| **Snord 34** | TCTGTGATGTTCTGCTATTACCTA | CAGTGGGGTTTTCATGAGGC |
| **Gapdh** | GAACATCATCCCTGCATCCA | CCAGTGAGCTTCCCGTTCA |
| **Hprt1** | TTGCTCGAGATGTCATGAAGGA | AATGTAATCCAGGACGTCAGCAA |

**Supplementary Table S10** Primer sequences for qPCR designed using Primer-BLAST.
